# *In vitro* remineralization of primary teeth with a mineralization-promoting peptide containing dental varnish

**DOI:** 10.1590/1678-7757-2020-0259

**Published:** 2020-09-07

**Authors:** Fatih TULUMBACI, Mustafa GUNGORMUS

**Affiliations:** 1 Ankara Yildirim Beyazit University School of Dentistry Department of Pediatric Dentistry Ankara Turkey Ankara Yildirim Beyazit University, School of Dentistry, Department of Pediatric Dentistry, Ankara, Turkey.; 2 Ankara Yildirim Beyazit University School of Dentistry Department of Basic Sciences Ankara Turkey Ankara Yildirim Beyazit University, School of Dentistry, Department of Basic Sciences, Ankara, Turkey.; 3 Ankara Yildirim Beyazit University School of Engineering and Natural Sciences, Biomedical Engineering Ankara Turkey Ankara Yildirim Beyazit University, School of Engineering and Natural Sciences, Biomedical Engineering, Ankara, Turkey.

**Keywords:** Remineralization, Mineralization-promoting peptide, Varnish, PLM, SEM

## Abstract

Mineralization-promoting peptides are attractive candidates for new remineralization systems. In previous studies, peptides have been applied as aqueous solutions, which is not a clinically relevant form.

**Objective:**

This study aims to investigate the efficiency of a mineralization-promoting peptide, applied in varnish, on remineralizing artificial caries on primary teeth.

**Methodology:**

55 primary molars were collected. Specimens were immersed in a demineralizing solution for 7 days and then, divided into 7 groups: Baseline: No-remineralization, Placebo: Blank colophony, F: Colophony 5% fluoride, P: Colophony 10% peptide, P+F: Colophony 5% fluoride and 10% peptide, Embrace: Embrace™ varnish, Durashield: Durashield™ varnish. A mixture of 35% w/v colophony varnishes were prepared in ethanol and applied accordingly. Specimens were immersed in a remineralization solution for 4 weeks and it was evaluated using PLM and SEM. Lesion depth reduction was examined by one-way ANOVA.

**Results:**

There was no significant difference in mean lesion depths between baseline (147.04 ± 10.18 µm) and placebo groups (139.73 ± 14.92 µm), between F (120.95 ± 12.23 µm) and Durashield (113.47 ± 14.36 µm) groups and between P (81.79 ± 23.15 µm) and Embrace (90.26 ± 17.72 µm) groups. Lesion depth for the P+F group (66.95±10.59 µm) was significantly higher compared to all other groups. All groups contained samples with subsurface demineralized regions. Number of subsurface demineralized regions were higher in fluoride-containing groups.

**Conclusions:**

We conclude that the mineralization-promoting peptide (MPP3) is effective in this *in vitro* study and the peptide shows benefits over fluoride as it yields less subsurface demineralized regions.

## Introduction

Caries formation is a result of a shift towards demineralization in the demineralization / remineralization cycle. When demineralization becomes dominant, carious lesions appear in certain teeth regions.^1^ Epidemiological data indicate that dental caries is the single most common chronic childhood disease, and its prevalence is increasing in most industrialized countries, with a 60 to 90% prevalence among children in school age.^2^

The demineralization process involves dissolution of hydroxyapatite, mainly caused by acidic attacks originating from the fermentation of sugars by microbial biofilm, leaving a demineralized lesion. Demineralized lesions usually turn into subsurface lesions by rapid remineralization of the top of the lesion in the presence of fluoride in saliva.^1^ These demineralized lesions without visible cavitation are defined as white spot lesions or incipient caries. Minimally invasive approaches, such as remineralization therapies, for treating incipient lesions, are of great significance in modern pediatric dentistry.^3^ Remineralization occur when the lost tooth mineral is recovered by the precipitation of soluble ions in saliva or biofilm. However, for remineralization to certain conditions must be satisfied. Firstly, underlying reasons causing demineralization, such as bacterial load and high dietary sugar and acid, should be eliminated. Furthermore, favorable conditions for remineralization, such as sufficient calcium and phosphate concentration, increased oral pH, agents that promote remineralization, should be provided.^3^

Until this date, fluoride has been the most widely used agent to prevent and to treat incipient caries. Fluoride works by partially replacing hydroxyl ions in the hydroxyapatite and thus forming fluorapatite, a more acid-resistant mineral.^4^ Due to the decades-long evidence on its caries-preventive effects, use of fluoride is recommended by the regulatory and scientific bodies. The doses used in dental applications does not pose a risk of acute poisoning. However, prolonged exposure to low doses has been shown to cause dental and skeletal fluorosis,^5^ development of subsurface lesions^6-9^ and change in enzymes activities, such as casein kinase II and alkaline phosphatase.^10^

Non-fluoride containing remineralization products are also available in the market. These products mostly act by increasing the soluble calcium and phosphate ions in the saliva via stabilized forms of metastable calcium phosphate phases. Upon contact with saliva, metastable calcium phosphate is dissolved, and calcium and phosphate ions are released to the oral cavity.^3^ However, clinical and *in vitro* studies report that increasing intra-oral calcium and phosphate concentration alone does not provide better results compared to standard oral hygiene regimes.^11,12^Therefore, there is still a need for new remineralization systems especially for pediatric population, which is more sensitive to fluoride.

Peptide agents that promote peptide mineralization have emerged as attractive candidates for such new remineralization systems.^13-16^Several peptides have been proposed for improving remineralization. Acidic fragments of naturally occurring biomineralization proteins or peptides designed or selected via experimental and computational tools have been shown to promote hydroxyapatite mineralization, therefore, they are suggested as agents for remineralizing the enamel. Self-assembling peptides have also been proposed as scaffolds to control the orientation of newly formed mineral, to resemble natural 3D architecture of the enamel.^17,18^

*In vitro*^15,19^and *in vivo*^20^ studies have yielded promising results for the use of peptides as remineralizing agents in dentistry. However, in these studies peptides have been applied as aqueous solutions. Aqueous solutions are challenging in a clinical setting as they require more patient’s cooperation and they do not provide slow continuous release.^21,22^The potential of these peptide agents has not yet been investigated as a real or prototype product that is more relevant to a clinical setting. To the best of our knowledge, no studies have been conducted using mineralization-promoting peptides in a real or prototype product format, especially focusing on primary teeth. This *in vitro* study aims to assess whether a mineralization-promoting peptide, applied in a colophony varnish preparation, is effective on remineralizing artificial carious lesions formed on primary teeth. We have compared the efficacy of peptide-containing, in-lab made varnishes, with two commercially available varnishes.

## Methodology

**Figure 1:**
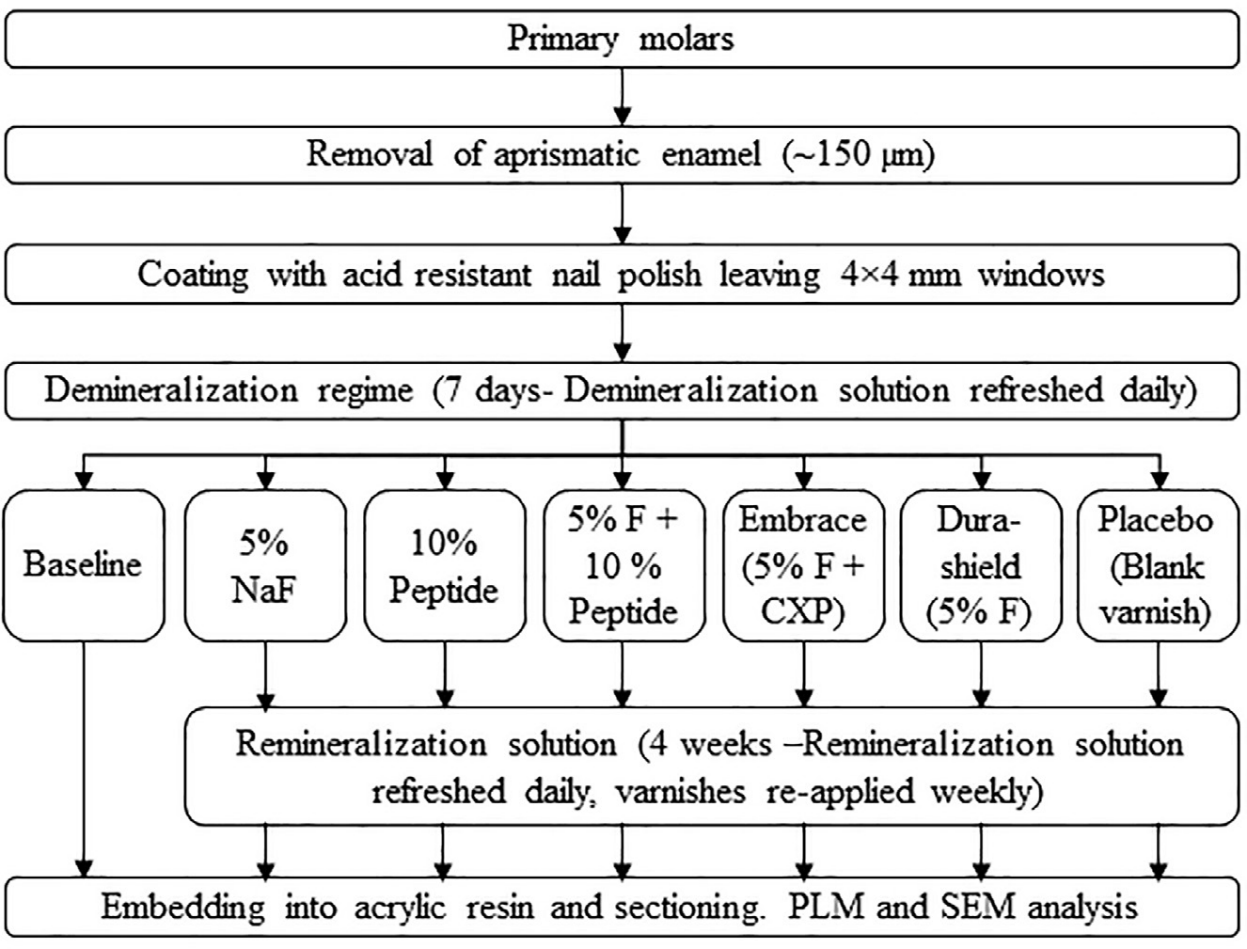
Outline of the study design

This study was approved by the “Research Ethics Committee (File number: 2017/55) and it has been conducted in accordance with the Declaration of Helsinki. All parents/guardians of the patients have signed a consent form following a written and verbal explanation of the study. A general outline of the study design is shown in Figure 1.

Collection of the teeth**:** An a *priori* power analysis was conducted using G*power to test the difference between seven independent group means using one-way ANOVA test, medium effect size of d=0.50 based on similar studies^19,23^ and an alpha value of 0.05. Result showed that a total sample size of n=91 with seven equal sized groups (n=13) was required to achieve a power of 0.80. Two additional samples were included in each group, resulting in seven equal sized groups of n=15.

In total, 55 primary molars, extracted due to exfoliation or orthodontic reasons from children aged between six and 12 years, were collected at the Pediatric Dentistry Clinic, Tepebasi Oral and Dental Health Hospital, Ankara Yildirim Beyazit University. The teeth were air dried by an air syringe and visually examined. Teeth containing visible structural defects, cavitated lesions, visible white-spot lesions and/or discoloration were excluded. After collection, soft tissue residues on the teeth were gently curetted, and cleaned with fluoride-free pumice, polishing brush, and non-fluoridated water. Samples were kept in 0.10% thymol solution (ADR Group, Istanbul, Turkey) until the time of use.

Preparation of the varnishes: The peptide used in this study (PGEKADRAEKADRA)^24^ was synthesized commercially at >98% purity (GenScript Biotech Corp. NJ, USA). Natural colophony was used to prepare the peptide-containing varnishes. Solid colophony was ground in a mortar to fine powder. The colophony powder was dissolved in ethanol (Sigma-Aldrich, MO, USA) to a 35% w/v concentration by heating up to 60°C and rigorous mixing. Three different varnishes were made in-lab: Colophony varnish with 5% NaF (F) (Merck & Co., NJ, USA), with 10% peptide (P), and with 5% NaF + 10% peptide (P+F). The peptide concentration was based on previous studies using similar peptides.^15,24^ Peptide and NaF were dissolved in ethanol and added to the colophony. A placebo varnish was prepared with blank colophony, containing no peptide or NaF. Two commercially available varnishes were used as control groups; Durashield (5% fluoride) (York, PA, USA) and Embrace (5% fluoride + Xylitol-coated Calcium and Phosphate (CXP)) (Pulpdent Corp., Watertown, MA, USA).

Formation of artificial caries: The crowns were cut at the cementoenamel junction using a Micracut 201 precision cutter (Metkon, Bursa, Turkey) under water cooling. The crowns were cut in mesiodistal direction to obtain two specimens from each crown. Each specimen was embedded in acrylic block (Orthocryl EQ, Dentaurum, Ispringen, Germany) with the enamel surface facing outward. Approximately 150 µm enamel layer was removed via serial polishing with 500, 1200, 2400, and 4000 grit silicon carbide polishing strips. Enamel surfaces were covered with 4×4 mm pieces of adhesive tape and the areas left open were coated with two layers of acid-resistant nail polish (Flormar MATTE, Kocaeli, Turkey). The artificial caries were formed by immersing the specimens in a demineralizing solution containing 2.20 mM Ca(NO_3_)_2_, 2.20 mM KH_2_PO_4_, 0.10 ppm NaF, and 50 mM acetic acid for seven days at 37°C, as described by Featherstone, Duncan, and Cutress^25^ (1979). All specimens were demineralized in the same container and the demineralization solution was refreshed daily.

Application of varnishes: Demineralized specimens were randomly distributed among seven groups. No varnish was applied on the baseline group. In the other groups, varnishes were applied on the exposed enamel surfaces using the applicators that came with the commercial varnishes and they were left to dry for two minutes in air.

Remineralization regime: The baseline group was not subjected to remineralization regime after demineralization. For other groups, each specimen was immersed into a 30 ml remineralization solution containing 1.50 mM CaCl_2_ (Merck & Co., NJ, USA), 0.90 mM KH_2_PO_4_ (Merck & Co., NJ, USA), 130 mM KCl (Merck & Co., NJ, USA), and 20 mM Tris-HCl (Sigma-Aldrich, MO, USA) (pH 7.0) in individual containers. The specimens were kept in the remineralization solution for four weeks.^26,27^The remineralization solution was refreshed daily and the varnishes were renewed weekly.^25^

Polarized Light Microscopy (PLM) Analysis: After the remineralization regime, varnishes on the specimens were scalped by a surgical knife. The specimens were embedded in acrylic resin (Orthocryl EQ, Dentaurum, Ispringen, Germany) and approximately 200 µm slices were cut via a Micracut 201 precision cutter (Metkon, Bursa, Turkey). The slices were further ground down to approximately 100 µm manually using 500 grit silicon carbide polishing strips and polished using 1200, 2400, and 4000 grit polishing strips. PLM analysis was carried out using a BA310 POL Trinocular microscope (Motic, Kowloon, Hong Kong) equipped with a Moticam 5 digital camera (Motic, Kowloon, Hong Kong) under 400X magnification. The lesion depths were measured using Image Focus 4.0 (Motic, Kowloon, Hong Kong). The measurements were performed by a pediatric dentist and a non-clinician researcher, both blind to the coding of the samples. The depths of lesions were measured at the cervical, central, and coronal regions and these depths were averaged. The percent reduction in lesion depth was estimated relative to the mean lesion depth measured on the Baseline group.

Scanning Electron Microscopy (SEM) Analysis: The specimens used for PLM analysis were placed on a carbon tape attached to an aluminum sample holder and coated with platinum using a Leica EM ACE200 vacuum coater (Leica Microsystems GmbH, Wetzlar, Germany). SEM imaging and Energy-dispersive X-ray spectroscopy (EDS) on the lesions sites were performed using a Hitachi SU5000 field emission SEM (Hitachi High Technologies Corp., Tokyo, Japan) at 10 kV accelerating voltage.

### Statistical Analysis

The inter-observer variability for lesion depth measurements was evaluated by intraclass correlation coefficient (ICC) using two-way mixed model, average measures, and absolute agreement. The difference in lesion depths were analyzed using One-Way ANOVA with Tukey’s post hoc test for multiple comparison. All statistical analyses were performed using IBM SPSS v25 (IBM Corp., Armonk, NY, USA) and the level of significance was set to α=0.05 for all analyses.

## Results

**Figure 2:**
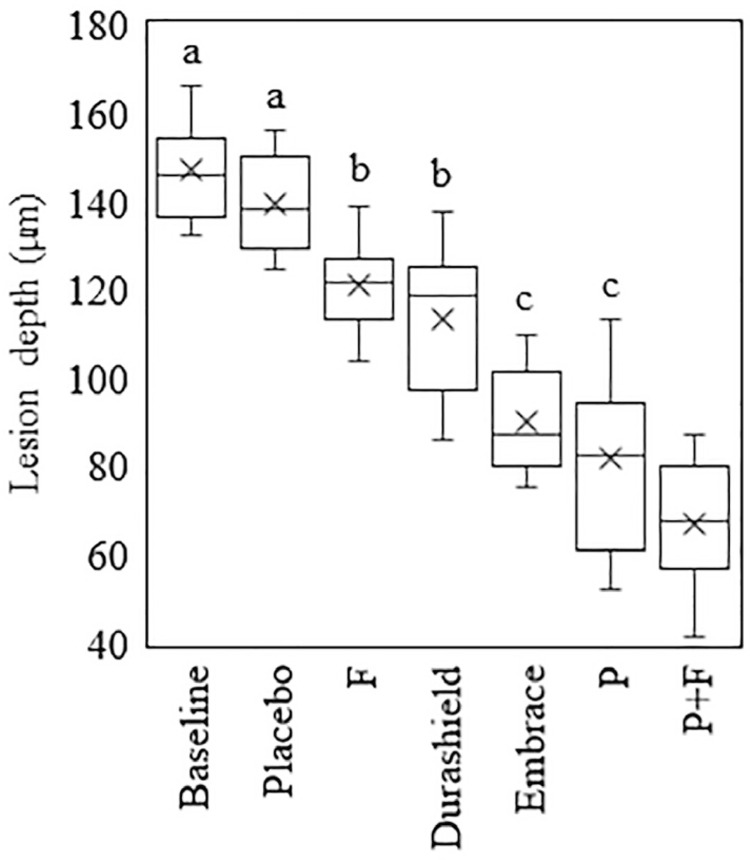
Mean lesion depths based on PLM images (Same letters indicate no statistically significant difference)

**Figure 3:**
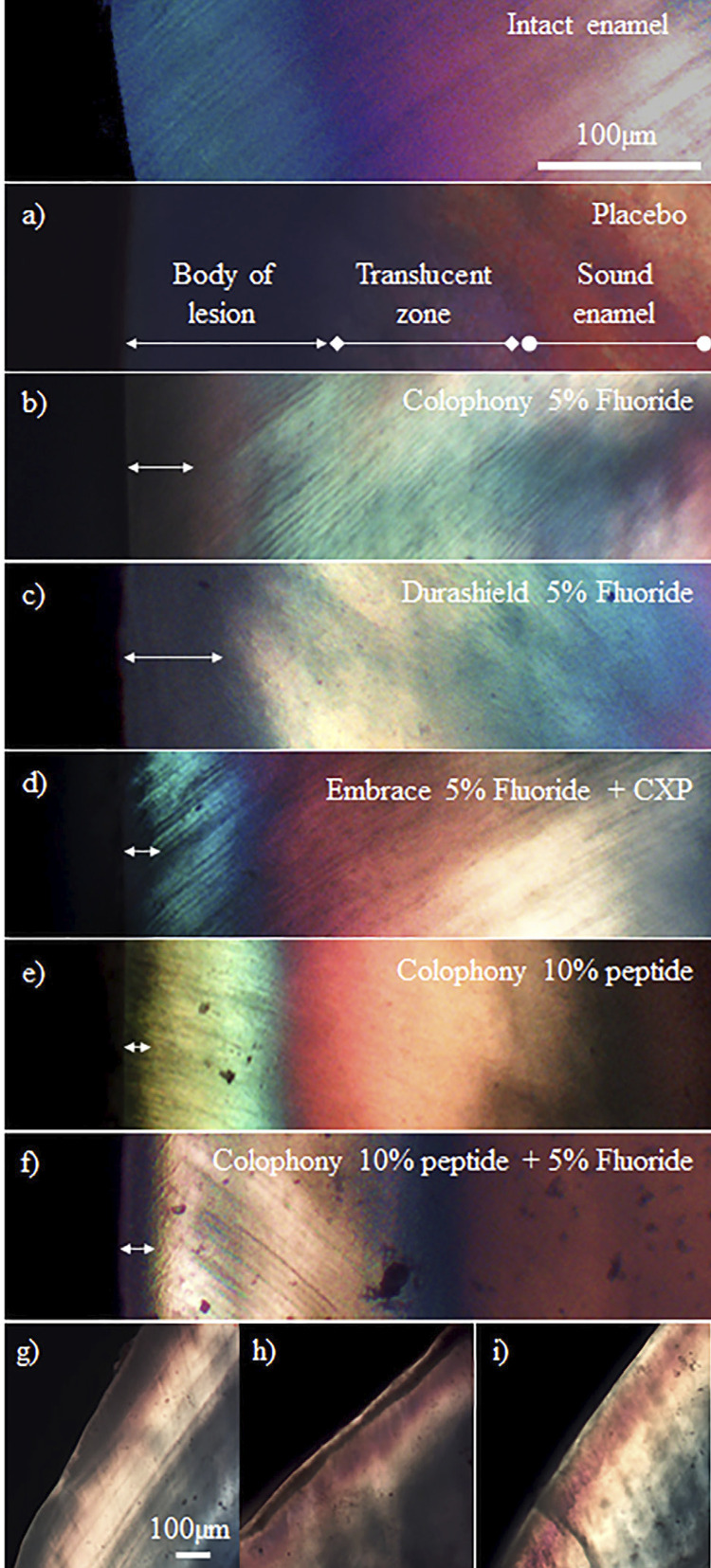
(a-f) Representative PLM images used for measuring the lesion depth decrease (g-i): Representative PLM images of subsurface demineralized regions

PLM Analysis: ICC showed an excellent degree of reliability between PLM measurements of the two raters. The average value of ICC was 0.989 with 95% confidence interval from 0.742 to 0.997 (F(104,104)= 255.217, p<0.001). One-Way ANOVA presented significant differences in percent lesion depth reduction among groups (F(5, 64) =32.855, p<0.001). Post-hoc comparison using Tukey HSD test showed that there was no significant difference in mean lesion depths between baseline (147.04 ± 10.18 µm) and placebo group (139.73 ± 14.92 µm), between F (120.95 ± 12.23 µm) and Durashield (113.47 ± 14.36 µm) groups and between P (81.79 ± 23.15 µm) and Embrace (90.26 ± 17.72 µm) groups. Lesion depth for the P+F group (66.95±10.59 µm) was significantly higher compared to all other groups (Figure 2). Representative PLM images of the specimens are shown in Figure 3a-f.

**Figure 4:**
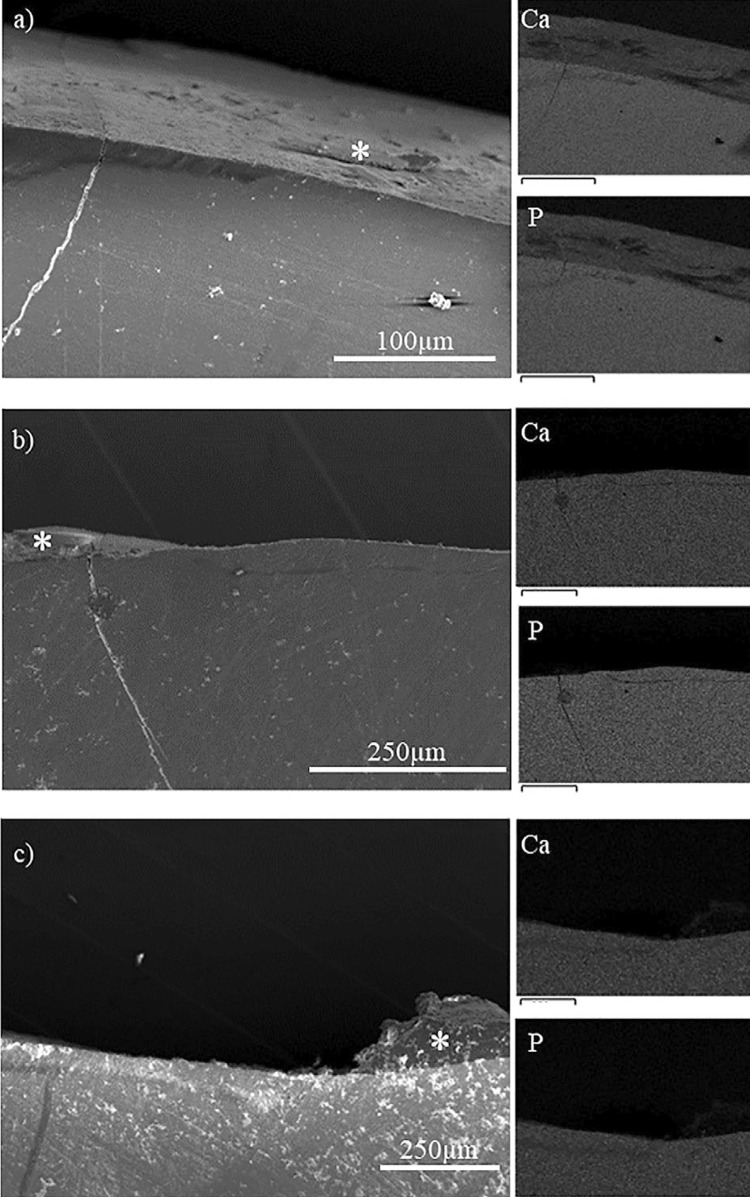
SEM images and the corresponding elemental mapping for calcium and phosphate showing subsurface demineralized regions in a) Durashield, b) F and c) P+F groups. (*: acid resistant film)

**Figure 5:**
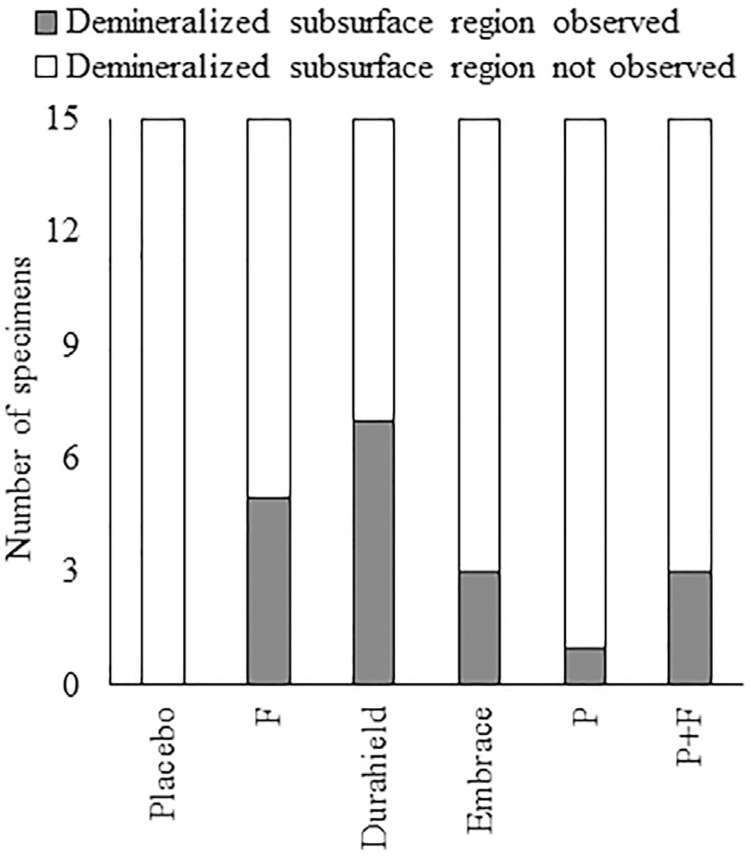
Number of specimens with subsurface demineralized regions in the groups

SEM Analysis: Overall morphology and elemental composition of the samples were investigated in the SEM analysis. SEM analysis, in parallel with PLM analysis, showed evidence of rapid surface remineralization leaving demineralized subsurface regions. (Figure 4) None of the groups were free of demineralized subsurface regions. Number of specimens in which demineralized subsurface regions were observed were highest in Durashield and F groups, followed by Embrace and P+F and lowest in the P group. (Figure 5) Example PLM images of subsurface demineralized regions are also shown in Figure 3g-i. In the EDS analysis collected from the lesion sites of specimens, fluoride was detected in the groups treated with fluoride-containing varnish.

## Discussion

Non-cavitated demineralization lesions restricted to the enamel represent the first stage of caries formation. In these lesions, enamel becomes more porous as the dissolution of mineral disrupts its natural architecture. However, these lesions are reversible by creating a suitable environment. Remineralization therapies aim to regain the lost mineral content by precipitating calcium and phosphate either available in the saliva or from external sources into the demineralized pores.^3^

The enamel thickness in primary teeth is nearly half the size of permanent teeth and the mineral content is lower compared to permanent teeth, resulting in weaker mechanical properties compared to permanent teeth.^26^ Therefore, the conditions for the restorative or remineralization therapies for primary teeth differ from those of permanent teeth.^28^ In this study, we have investigated the efficacy of a mineralization-promoting peptide in a varnish on remineralizing artificial lesions of primary teeth using *in vitro* model. We have shown that the peptide, when used alone performs comparable to a commercial varnish containing fluoride and bioactive particles and it performs better compared to a commercial varnish containing only fluoride. When used in combination with fluoride, the peptide performs better compared to both commercial varnishes in terms of lesion depth reduction.

*In vitro* demineralization / remineralization models have been used extensively to study the potential of proposed remineralization agents. In this study, we have used a model described by Featherstone, Duncan and Cutress^25^ (1979). This model aims to mimic the natural demineralization and remineralization cycle that occur with teeth.

PLM is widely used to assess remineralization both in *in vitro* and *in vivo* studies.^29-32^It relies on the principle that light is diffracted positively when passing through porous and organic materials and it is diffracted negatively when passing through crystalline and inorganic materials. Therefore, sound and demineralized enamel is easily distinguished under PLM. SEM is also useful to determine the elemental composition and the overall morphology of enamel after remineralization. Other methods to assess remineralization include microradiography^31^, microhardness measurements,^33^ laser fluorescence (LF),^34^ quantitative light-induced fluorescence (QLF)^35^ or using precursor ions of radioactive isotopes.^18^ These methods are complementary and they provide information about different aspects of remineralization. For example, LF and QLF provide rapid information on the presence and severity of lesions but LF requires presence of bacterial toxins to detect lesions and it is not suitable for most *in vitro* models.^34^ On the other hand, QLF does not require bacterial toxins do detect caries, but it requires expensive equipment.^35^ Microhardness measurements provide information from relatively small sections of the specimens, but it exclusively provide information about mechanical properties. On the other hand, microradiography can provide volumetric and density data on the whole specimen. In our study we have used a combination of PLM and SEM. Subsurface demineralized regions are not easily identified in secondary electron imaging, however, in combination with PLM and elemental mapping by EDS, the subsurface demineralized regions were investigated.

The potential of mineralization-promoting peptides in dentistry has been investigated extensively and many different peptides have been identified with promising results.^13-16^ In these studies, peptides have been used as aqueous solutions, which is not an ideal form for clinical application. Vieira, Ruben and Huysmans^22^ (2005) have compared the effect of a fluoride gel, aqueous fluoride, and fluoride varnish on enamel erosion *in vitro*. They have shown that among the three tested compounds, fluoride varnish results in significantly lower calcium loss and erosion. Gel and aqueous forms result in similar and higher calcium loss and erosion.^22^ Eakle, et al.^21^ (2004) have investigated the salivary fluoride levels following the application of fluoride as rinse or varnish. These authors have shown that salivary fluoride levels return to baseline two hours after applying the fluoride rinse, whereas the levels remain elevated for an average of 24 hours after a fluoride varnish is applied.^21^ Gel and foam fluoride formulations are also available; however, varnish is the recommended form of topical fluoride application for children under 6.^36,37^Therefore, we have devised a simple colophony-based varnish, containing the peptide to assess the potential of a mineralization-promoting peptide under conditions more relevant to clinic. Colophony was chosen in this study due to its relative simplicity, ease of use and affordability. Colophony is hydrophobic and it does not dissolve in saliva. Colophony can be dissolved in non-polar solvents, such as alcohol, and when it is applied into a surface, it re-solidifies by alcohol evaporation.^38,39^ When re-solidification of colophony is considered, isopropyl alcohol is a more suitable solvent as it evaporates faster compared to ethanol. However, since the peptide used in this study requires a polar solvent, ethanol was chosen considering that it can dissolve both polar and non-polar compounds. The lyophilized peptides are not readily dissolvable in commercial varnishes. They must be dissolved in ethanol first and then added into the varnishes. The addition of extra ethanol would dilute the commercial varnishes and change their composition. Therefore, we opted to prepare in-lab made varnishes with the peptide, rather than adding the peptides directly to the commercial varnishes. In the in-lab made varnishes, the starting amount of the varnish was adjusted to compensate the additional peptide added in ethanol.

Durashield and Embrace are also colophony-based varnishes that are composed of the same amount of fluoride with the F group. Embrace varnish includes xylitol coated calcium phosphate (CXP^TM^) in addition to fluoride. When compared with the F group, no statistically significant differences in remineralization were observed in the Durashield group. This indicates that our varnish preparation carried out its intended function. Embrace varnish resulted in higher remineralization compared to Durashield. It is known that the amount of available calcium and phosphate ions significantly influences the efficiency of fluoride in remineralization.^40^ Our results confirm this notion, indicating that extra calcium and phosphate ions provided by the CXP^TM^ in Embrace varnish increases the efficiency of the remineralization.

No statistically significant differences in remineralization were observed between the Embrace varnish and the P group. This indicates that the mineralization-promoting effect of the peptide may compensate the absence of external calcium and phosphate by promoting the precipitation of the existing calcium and phosphate. This observation may be explained by the effects of fluoride and macromolecules on the precipitation kinetics of calcium phosphate minerals. Besides producing more acid-resistant fluorapatite mineral, fluoride can also increase HAp formation kinetics by increasing the hydrolysis of the precursor phase octacalcium phosphate (OCP) into HAp.^41^ Peptides or proteins can also increase HAp formation, mainly by acting as nucleation sites.^42^ In Durashield and the F groups, which had the same amount of fluoride, we observed a similar reduction in the lesion depth, due to the promoting effect of fluoride. In the Embrace group, reduction in lesion depth was higher compared to F or Durashield possibly due to external calcium and phosphate in the formulation. There was no fluoride in the P group, however, the accelerating effect of the peptide resulted in a similar reduction of the lesion depth compared to fluoride combined with external calcium and phosphate.

Lastly, the highest remineralization was observed in the P+F group. The increase in remineralization in the P+F group was not drastic compared to the P or Embrace groups, but it is statistically significant. This is possibly due to the synergistic effect of different mechanisms that promote HAp formation by both fluoride and peptide. Future studies combining the mineralization-promoting peptide, fluoride, and external of calcium and phosphate ions may support the identification of more effective formula with superior remineralization capabilities.

It should be noted that, although fluoride containing varnishes resulted in higher remineralization rates, the occurrence of demineralized subsurface regions was also higher with these varnishes. Demineralized subsurface regions are the result of rapid remineralization near the surface of the lesion, preventing the ions to penetrate deeper into the lesion. Rapid superficial remineralization by fluoride is also associated with the formation of demineralized subsurface regions.^4^ The P group, which contains the peptide, but does not contain fluoride, resulted in the least number of demineralized subsurface regions. Although the combination of the peptide and the fluoride (P+F group) resulted in the highest reduction in lesion depth, more subsurface lesions were observed compared to the P group. The peptide used in this study is proposed to act by attracting the available calcium and phosphate ions, thus, creating local supersaturation zones.^24^ In contrast, existing products provide the fluoride (and calcium and phosphate) to a relatively larger area within the lesion, which may explain the higher number of subsurface lesions observed.

This study is limited by the inherent limitations of *in vitro* demineralization/remineralization models, i.e.; lack of dynamic environment of *in vivo* conditions, lack of pellicle and biofilm on the tooth surface, and difficulty in matching the solid/solution ratios occurring *in vivo*.^43^ Another limitation is the simple formulation of in-lab made resin. The formulations of commercial resins are usually optimized to improve their performance and they are manufactured under GMP conditions. We have used a commercially available unmodified colophony resin as purchased. Therefore, there is room for optimization and improvement in the in-lab made varnish formulation. An *in vivo* rat caries model using the same peptide-containing varnish has been concluded and it is being prepared for publication. Future *in vivo* or *in situ* studies combining the mineralization-promoting peptide, fluoride, and ion releasing bioactive particles may help identifying a more effective formulation with superior remineralization capabilities.

## Conclusions

Within the limitations of this *in vitro* study, we conclude that MPP3 is effective in varnish formulations for remineralization of enamel lesions. We also conclude the peptide-containing varnish shows benefits over fluoride-containing varnish as it results in less demineralized subsurface regions.
